# Hybrid Vision Transformer–CNN Framework for Alzheimer’s Disease Cell Type Classification: A Comparative Study with Vision–Language Models

**DOI:** 10.3390/jimaging12030098

**Published:** 2026-02-25

**Authors:** Md Easin Hasan, Md Tahmid Hasan Fuad, Omar Sharif, Amy Wagler

**Affiliations:** 1 Department of Mathematical Sciences, The University of Texas at El Paso, El Paso, TX 79968, USA; 2 Department of Computer Science and Engineering, University of Manitoba, Winnipeg, MB R3T 2N2, Canada; 3 School of Mathematical and Statistical Science, The University of Texas at Rio Grande Valley, Edinburg, TX 78541, USA; 4 Office of Research, Creativity, and Economic Development, New Mexico State University, Las Cruces, NM 88003, USA

**Keywords:** image classification, vision transformer–convolutional neural network, multimodal vision–language models, transfer learning, Alzheimer’s disease

## Abstract

Accurate identification of Alzheimer’s disease (AD)-related cellular characteristics from microscopy images is essential for understanding neurodegenerative mechanisms at the cellular level. While most computational approaches focus on macroscopic neuroimaging modalities, cell type classification from microscopy remains relatively underexplored. In this study, we propose a hybrid vision transformer–convolutional neural network (ViT–CNN) framework that integrates DeiT-Small and EfficientNet-B7 to classify three AD-related cell types—astrocytes, cortical neurons, and SH-SY5Y neuroblastoma cells—from phase-contrast microscopy images. We perform a comparative evaluation against conventional CNN architectures (DenseNet, ResNet, InceptionNet, and MobileNet) and prompt-based multimodal vision–language models (GPT-5, GPT-4o, and Gemini 2.5-Flash) using zero-shot, few-shot, and chain-of-thought prompting. Experiments conducted with stratified fivefold cross-validation show that the proposed hybrid model achieves a test accuracy of 61.03% and a macro F1 score of 61.85, outperforming standalone CNN baselines and prompt-only LLM approaches under data-limited conditions. These results suggest that combining convolutional inductive biases with transformer-based global context modeling can improve generalization for cellular microscopy classification. While constrained by dataset size and scope, this work serves as a proof of concept and highlights promising directions for future research in domain-specific pretraining, multimodal data integration, and explainable AI for AD-related cellular analysis.

## 1. Introduction

Alzheimer’s disease (AD) is a neurodegenerative disorder caused by the accumulation of misfolded amyloid-beta (αβ) protein, which severely impacts the brain and central nervous system, leading to the progressive loss of memory and the ability to function independently. Despite numerous hypotheses regarding the origins and causes of AD, current medical treatments are unable to cure most of these conditions [1,2]. Over the past three decades, the pathology of Alzheimer’s disease (AD) has been extensively studied through the key pathological features of AD, including the accumulation of proteins, the loss of neurons and synapses, and changes associated with reactive processes [3].

The prevalence of AD continues to grow, with approximately 6 million individuals affected in the United States alone. This number is expected to rise to 12.7 million by 2050, driven by an aging population and the absence of major breakthroughs in prevention or treatment [4]. The ability to delineate specific brain cell types in AD pathology is critical for understanding the cellular mechanisms that contribute to disease progression and developing targeted therapeutic strategies [5]. However, studying the interactions and adaptability of different brain cell types in living organisms remains a significant challenge, particularly when investigating large-scale structural brain changes linked to physiological and pathological states [6,7].

Artificial intelligence (AI) has emerged as a transformative tool in biomedical imaging, providing advanced analytical frameworks to support early diagnosis and mechanistic understanding of diseases. While AI-based models have achieved remarkable success in detecting AD stages from magnetic resonance imaging (MRI) data—such as the convolutional neural network (CNN)-based approach by Alshammari et al., which achieved 97% accuracy across four AD stages [8]—the classification of AD-associated cell types remains an underexplored but critical frontier. Accurate identification of cellular phenotypes can reveal insights into disease progression at the microscopic level and support the discovery of novel drug targets.

The classification of key cell types involved in AD, such as astrocytes, cortical neurons, and SH-SY5Y neuroblastoma cells, using computer vision and AI techniques offers great potential for advancing therapeutic research [9,10]. Several state-of-the-art deep learning architectures have shown promise in AD-related image analysis. For instance, DenseNet [11], which employs dense connectivity to promote feature reuse, achieved an 86% classification accuracy in AD MRI categorization [12]. Similarly, ResNet’s residual learning framework effectively mitigates vanishing gradient problems, with pretrained ResNet-50 models achieving up to 98.99% accuracy in AD MRI classification [13,14]. MobileNet and InceptionNet architectures have also been leveraged for efficient AD diagnosis. MobileNet’s depthwise separable convolutions optimize computational costs while maintaining accuracy, yielding 94% accuracy in AD classification [15,16], whereas InceptionNet’s multi-scale design enhances feature extraction and performance with minimal resource overhead [17,18].

Furthermore, EfficientNet models overcome conventional CNN limitations by uniformly scaling the network depth, width, and resolution, thereby achieving superior performance with reduced manual tuning. Sethi et al. [19] demonstrated that by employing a composite scaling coefficient to jointly adjust these parameters, the EfficientNet transfer learning (TL) model effectively mitigates architectural imbalance and enhances representational efficiency. The model is characterized by two guiding principles: first, an optimized baseline architecture that accelerates convergence during training, and second, a compound scaling strategy that maintains accuracy while deepening the network. Its design relies on 2D depthwise separable convolutions composed of multiple inverted mobile bottleneck blocks, which contribute to computational efficiency and improved feature extraction. One of EfficientNet’s major advantages over conventional CNNs lies in this systematic scaling approach, which employs predefined scaling factors to balance the model capacity and computational costs. Consequently, the EfficientNet TL strategy has been successfully applied for Alzheimer’s disease (AD) diagnosis, achieving 91.36% accuracy and an AUC of 83% on the ADNI dataset [19]. Similarly, Zheng et al. [20] proposed a modified 3D EfficientNet architecture that addresses the limitations of conventional scaling methods by introducing a max pooling layer after the initial convolution, ReLU activation outside the main building block, and replacing the global average pooling layer with three fully connected layers. These enhancements led to substantial performance gains, achieving 83.33% classification accuracy and 88.75% AUC, an improvement of more than 10% over the original EfficientNet model.

Despite these advancements, EfficientNet-based architectures remain limited by their reliance on large-scale pretraining datasets such as ImageNet, which may introduce domain bias when applied to biomedical images. Future research should focus on domain-specific pretraining and adaptive scaling strategies tailored to medical and cellular imaging data.

Astrocytes, cortical neurons, and SH-SY5Y cells each play critical roles in AD pathology. Astrocytes regulate key neuropathological processes, cortical neurons experience significant atrophy, and SH-SY5Y cells undergo structural and molecular alterations linked to AD progression [21,22,23]. Leveraging AI-driven deep learning frameworks to classify these cell types from microscopic images can provide valuable insights into disease mechanisms and inform drug development strategies [5,24,25].

**Figure 1:**
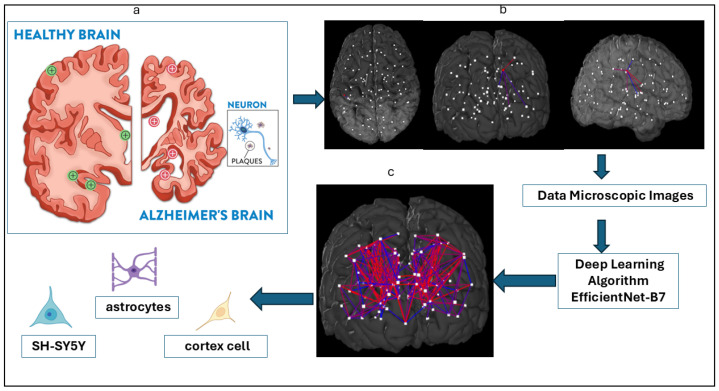
Study schematics of Alzheimer’s disease cell type detection. (**a**) Sketch of the brain and Alzheimer’s disease by the Fisher Center for Alzheimer’s Research Foundation. (**b**) Brain parcellation in sagittal, coronal, and axial views drawn from Budapest Reference Connectome version 3.0. (**c**) Classification of three key cell types—astrocytes, cortex cells, and SH-SY5Y cells—associated with AD using an AI-driven EfficientNet-B7 architecture applied to cell type-specific microscopic images.

While accurate cell classification is an essential step in addressing fundamental biological questions in AD research, challenges such as low image contrast, high cell density, and limited annotated data often hinder conventional analysis [7,26]. To address this gap, the present study focuses on three key AD-related cell types—astrocytes, cortical cells, and SH-SY5Y cells—using a hybrid transformer–CNN architecture built upon EfficientNet-B7. This model integrates convolutional and attention-based mechanisms to enhance feature extraction and improve generalization in multi-class cellular image classification. Figure 1 illustrates the overall schematic of our approach.

This study presents an advanced CNN-based hybrid architecture for classifying AD-associated cell types using transfer learning and attention-based enhancement. By improving the precision of cell type identification, our approach contributes to a deeper understanding of AD pathology and provides a foundation for developing targeted therapeutic interventions.

## 2. Related Work

### 2.1. CNN-Based Approaches

In this section, we review several CNN-based methods that have been used for image classification in the past and how they inform the proposed approach.

One highly relevant CNN-based model is DenseNet [11]. DenseNet has a distinct connectivity scheme because it adds direct links between any layer and successive layers. This allows the feature maps of all previous layers x0,…,x(l−1) to be sent to the *l*th layer. This scheme facilitates the extraction and sharing of feature maps throughout the network, which improves accuracy and speeds up inference times. Consequently, it is defined as follows: x(l)=H(l)([x0,x1,…,x(l−1)]), where [x0,x1,…,x(l−1)] denotes the combination of feature maps created in layers 0,…,l−1. The developers called this network architecture a “Dense Convolutional Network”, or DenseNet, because of its dense interconnectedness. The several inputs of H(l) in equation can be combined into a single tensor for simplicity of implementation [11].

Another relevant CNN-based model is ResNet [13], which considers H(x) as an underlying mapping that can be fitted by a few stacked layers, though not necessarily the entire network, with *x* representing the inputs to these layers, as in DenseNet. It is analogous to hypothesizing that numerous nonlinear layers can asymptotically approximate the residual functions; that is, H(x)−x (assuming that the input and output are of the same dimensions). Therefore, the layers explicitly approximate the residual function F(x)=H(x)−x instead of assuming the stacked layers to approach H(x). In this framework, the initial function is F(x)+x rather than H(x)−x. Both representations can provide an asymptotic approximation of the required functions; however, their respective learning curves may differ. From this perspective, it is possible to apply residual learning to every stacked level. Formally, they defined a building block as follows: $y=F(x,W_{i})+x$ [13]. In this case, the vectors *x* and *y* serve both as input and output for the layers being considered. The function $F(x,W_{i})$ serves as a representation of the residual mapping that must be learned. Then, using a skip connection and element-wise addition, the operation F+x is carried out. Note that in the equation $y=F(x,W_{i})+x$, the dimensions of *x* and *F* must be equivalent. In the case that they are not, a linear projection $W_{s}$ using the skip connections to fit the dimensions ($y=F\left(x,W_{i}\right)+W_{s}x$) [13] is a reasonable solution.

The MobileNet model is built upon depthwise separable convolutions, which are a kind of factorized convolution. This approach decomposes a standard convolution into two separate convolutions: a depth-wise and a point-wise convolution. In MobileNets, each input channel is assigned a single filter as a result of the use of depthwise convolution. The outputs of the depthwise convolution are combined with an additional 11 convolutions after the point-wise convolution. The inputs are filtered and combined using a typical convolution algorithm, which results in a new set of outputs. The factorization leads to a significant reduction in both the computational cost and model size. To apply a single filter to each input channel, the authors applied depth-wise convolutions (input depth), and the depth-wise layer’s output was then combined linearly using point-wise convolution. In both layers of MobileNets, batch normalization and ReLU nonlinearities are used [15].

Tan et al. [27] proposed EfficientNet, which is a new compound scaling method using a compound coefficient. The compound scaling of the network’s breadth, depth, and resolution are as follows:

$$\begin{array}{c} \mathrm{depth}:d=\alpha^{\phi }\\ \mathrm{width}:w=\beta^{\phi }\\ \mathrm{resolution}:r=\gamma^{\phi }\\ s.t.\alpha \beta^{2}\gamma^{2}\approx 2 \end{array}$$

where α≥1, β≥1, and γ≥1 [27]. Note that these can be identified using a simple grid search. A user-specified coefficient determines the availability of additional resources while indicating the assignment of the parameters for model scaling to the depth, width, and resolution of the network. In particular, the floating point operations per second (FLOPS) of a standard convolution operation are proportional to *d*, $w^{2}$, and $r^{2}$, or it is twice as deep as the network. Since convolution operations usually dominate the computational efficiency in ConvNets, scaling a ConvNet with the equation dependent on these parameters will increase the total FLOPS by approximately (α $\beta^{2}$ $\gamma^{2}{)}^{\phi }$ [27]. In our proposed method, we improved the pretrained EfficientNetB7 by adding seven ConvNets, batch normalizations, and L2 regularizations to the EfficientNetB7 to increase the model accuracy and reduce overfitting.

Recent studies have demonstrated the effectiveness of ML techniques in diagnosing Alzheimer’s disease (AD) using neuroimaging data such as magnetic resonance imaging (MRI) and positron emission tomography (PET). Fathi et al.[28] reviewed 74 studies on eight different databases, resulting in 736 studies on deep learning for early Alzheimer’s disease diagnosis. They found that CNN-based models outperform other deep learning models but lack explainability due to their black box nature. Moreover, most of the models focus on discriminative features for AD or MCI classification, improving performance but limiting interpretability. Additionally, they found there were 13 studies reporting this classification group for eight different databases, with the accuracy being in the range of 64.04–99.7% and the mean value being 87.43% [28].

Jain et al. [29] achieved 95.3% accuracy using a 2D-CNN on sMRI images from the ADNI dataset. Ding et al. [30] employed an Inception v3 CNN on PET scans, achieving 82% specificity and 100% sensitivity. Chitradevi et al. [31] utilized an AlexNet CNN on MRI images, reporting 95% accuracy, 94% specificity, and 95% sensitivity. Nawaz et al. [32] achieved the highest accuracy of 99.21% using SVMs on MRI data from the OASIS dataset. Kundaram et al. [33] reported 98.57% accuracy using a CNN on MRI images from ADNI. These studies highlight the potential of various deep learning architectures and traditional machine learning methods in AD diagnosis across different neuroimaging modalities and datasets. However, these studies were unable to validate their analyses with cell type-specific data, despite the fact that Alzheimer’s disease (AD) is characterized by neuronal death and tissue loss in the brain.

Several studies have addressed the challenge of data limitation in Alzheimer’s disease (AD) research using various approaches. Vu et al. [34] combined MRI and PET modalities from the ADNI dataset to increase data volume, albeit at the cost of increased computational complexity. Jain et al. [29] employed transfer learning to mitigate the scarcity of sMRI data, reducing computational costs. Salehi et al. [35] merged two ADNI MRI datasets to expand their sample size, though this approach may introduce inconsistencies in image formats and sizes. Basaia et al. [36] combined ADNI and Milan datasets during the testing phase, achieving higher accuracy in various classification tasks, except for AD vs. HC classification. Additionally, Khvostikov et al. utilized both structural MRI (sMRI) and diffusion tensor imaging (DTI) modalities from the ADNI dataset for Alzheimer’s disease classification, demonstrating that while the addition of DTI data can provide complementary information, it may also introduce noise in smaller datasets, highlighting the trade-offs in multi-modal approaches for neuroimaging analysis [37]. However, this method faced challenges in feature interpretability. These strategies demonstrate the trade-offs between data augmentation, computational complexity, and model performance in overcoming data limitations in AD research.

This review of the major CNN-based models—DenseNet, ResNet, MobileNet, and EfficientNet—highlights their unique architectural innovations and their application to AD diagnosis. Recent SOTA approaches have used multimodal data, hybrid architectures, and explainable AI to improve diagnostic performance. However, most studies lack validation on cell-type-specific data, despite AD’s cellular pathology involving astrocytes, cortical neurons, and SH-SY5Y cells. This limitation underscores the need for models tailored to cellular-level analysis. Our study addresses this gap by extending these architectures to classify AD-related cell types, bridging the divide between computational and cellular neuroscience.

### 2.2. Prompt-Based LLM

Recent research has extended instruction tuning and reasoning prompting from pure language models to vision–language and multimodal systems. For example, InstructBLIP [38] reformulates 26 public vision–language tasks into an instruction format and introduces an instruction-aware query transformer, achieving strong zero-shot performance across held-out datasets. Similarly, LLaVA uses machine-generated image-instruction pairs to train a vision encoder plus LLM that shows effective image-language chat and reasoning capabilities [39]. In the biomedical domain, MedCLIP [40] applies contrastive learning on unpaired medical image and text data and demonstrates zero-shot prediction gains on medical imaging tasks. These works show that while multimodal LLMs offer strong global reasoning and conversational abilities, they often require domain-specific adaptation (especially for fine-grained or domain-specialized tasks) and may still lag supervised models in highly specialized visual discrimination in Table 1. In this study, we therefore include prompt-based evaluations (zero shot, few shot, and chain of thought) of multimodal LLMs alongside our proposed hybrid ViT–CNN architecture to quantify this trade-off in the Alzheimer’s disease cell type classification setting.

## 3. Methodology

This study investigates Alzheimer’s cell classification from microscopy imagery through three complementary methodological paradigms: (1) deep learning-based visual modeling, (2) multi-modal large language model (LLM) reasoning via prompt-based evaluation, and (3) a proposed hybrid transformer–CNN model that integrates global semantic and fine-grained morphological information. In the following, a detailed description of the data is provided, followed by a summary of the baseline and proposed models that were evaluated on that data.

### 3.1. Data Preparation

**Figure 2:**
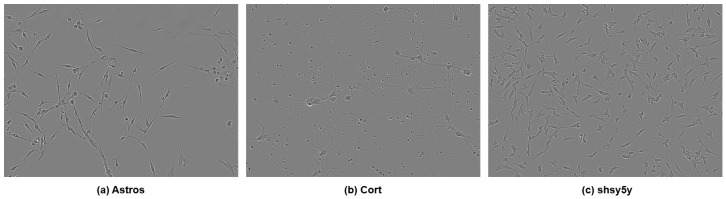
Three types of cells: (**a**) astrocyte, (**b**) cortical, and (**c**) SH-SY5Y.

The dataset consisted of 606 label-free phase-contrast microscopy images, including 320 cortical images, 150 SH-SY5Y images, and 130 astrocyte images. These were obtained from the Incucyte Live-Cell Analysis System [7]. The dataset exhibited substantial class imbalance, which poses challenges for supervised learning under limited sample regimes. In Figure 2, three types of cells are shown.

To address this imbalance, we employed the synthetic minority oversampling technique (SMOTE) [47] under a strictly controlled experimental protocol. Importantly, the SMOTE was applied exclusively to the training data within each cross-validation fold and never to the validation or test sets, thereby eliminating any possibility of data leakage during evaluation. Furthermore, the SMOTE was applied in feature space rather than pixel space. Specifically, deep feature embeddings were first extracted from pretrained backbone networks, and the SMOTE was used to interpolate between minority-class feature vectors. The resulting synthetic feature representations were then used only during model training. This approach avoids generating unrealistic pixel-level images while enabling controlled rebalancing of class distributions in representation space. After applying the SMOTE within the training folds, the effective training set size increased to 960 samples, while the validation and test sets remained composed solely of original, unmodified images. All reported test results therefore reflect performance on real microscopy data only.

Stratified fivefold cross-validation was employed to ensure consistent class proportions across splits, and identical preprocessing pipelines were applied across all folds. The training set facilitated the acquisition of knowledge by the model, the validation set aided in fine-tuning the model, and the test set offered an impartial assessment of the model’s performance.

#### Data Augmentation

Robust data augmentation was applied to enhance generalization and reduce overfitting. The augmentations include RandomResizedCrop, ColorJitter, GaussianBlur, and MixUp/CutMix regularization. These transformations simulate realistic variations in microscopy conditions while maintaining biological integrity.

### 3.2. Deep Learning Baseline Models

To establish strong and reliable baselines, we employed several widely adopted convolutional neural network (CNN) architectures pretrained on ImageNet, including ResNet-50, VGG16, DenseNet121, and EfficientNet-B7. These models were selected due to their proven effectiveness in medical image analysis and transfer learning scenarios.

All baseline networks followed a unified transfer learning framework. For each architecture, the original ImageNet classification head was removed, and the network was initialized with pretrained weights. The input images were processed using the model-specific preprocessing function to ensure consistency with the feature distribution learned during ImageNet pretraining. The pretrained backbone served as a feature extractor, generating high-level convolutional representations of the microscopy images. During fine-tuning, only the last portion of the backbone layers was updated, while earlier layers remained frozen. To maintain stable feature statistics under domain adaptation, all batch normalization layers within the backbone were kept frozen.

To adapt the pretrained backbones to the Alzheimer’s microscopy classification task, a lightweight task-specific classification head was appended. The extracted feature maps were first aggregated using global average pooling (GAP) to obtain a compact feature representation. This representation was passed through a fully connected layer, followed by batch normalization, ReLU activation, and dropout for regularization. A final fully connected layer with three output neurons and Softmax activation produced class probabilities for the three diagnostic categories.

**Figure 3:**
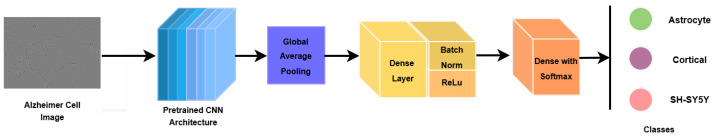
Deep learning model architectural pipeline.

Figure 3 illustrates this transfer learning architecture.

#### 3.2.1. Optimization and Training Set-Up for Deep Learning Models

- **Optimizer**: AdamW, known for stable convergence and adaptive weight decay.
- **Loss Function**: CrossEntropyLoss with class weighting to counter data imbalance.
- **Batch Size**: 32.
- **Epochs**: 50–100 (based on convergence criteria).

#### 3.2.2. Evaluation Objective for Deep Learning Models

These baseline CNN models act as a reference framework for evaluating both prompt-based and hybrid transformer–CNN models. The performance of each model was measured in terms of accuracy, macro F1 score, and class-wise confusion matrices.

### 3.3. Multi-Modal LLM Evaluation via Prompting

To examine the reasoning and interpretative capabilities of large-scale multimodal vision–language models on cellular microscopy images, we evaluated Gemini 2.5 Flash and GPT-5, both of which support joint visual and textual inputs. These models were assessed without gradient-based fine-tuning, relying exclusively on prompt engineering to perform the classification task. Consequently, the LLM results are intended to serve as *prompt-only baselines* rather than fully optimized vision classifiers.

All multimodal LLM experiments were conducted for **cell type classification** and not disease stage prediction. Each model was instructed to categorize microscopy images into one of three cell types: *astrocyte, cortical neuron, or SH-SY5Y neuroblastoma cells*. This formulation ensured consistency with the supervised deep learning and hybrid models evaluated in this study.

#### 3.3.1. LLM Prompting Strategies

We employed three complementary prompting paradigms to analyze how contextual guidance and explicit reasoning affect multimodal LLM performance. The exact prompt templates used in all experiments are reported below for reproducibility:

- **Zero-Shot Prompting:** The model is provided with a task instruction without labeled examples, evaluating its intrinsic visual reasoning capability.
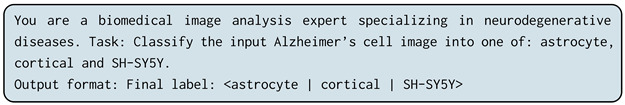
- **Few-Shot Prompting:** The prompt includes a small number (3–5) of labeled examples to provide contextual guidance via in-context learning.
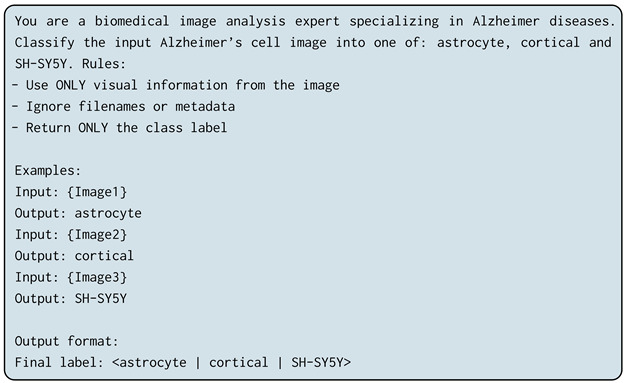
- **Chain-of-Thought (CoT) Prompting:** The model is explicitly instructed to reason through intermediate steps prior to classification, encouraging structured visual analysis similar to human expert reasoning.
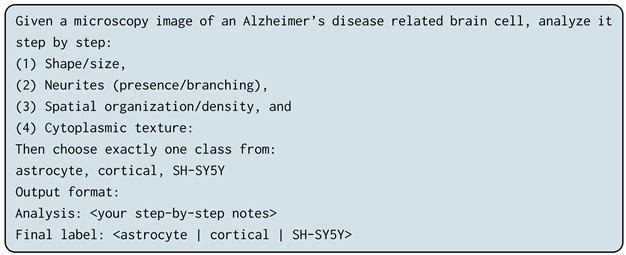

#### 3.3.2. Evaluation Procedure for LLM Models

Each multimodal LLM processes the same microscopy images under all three prompting configurations. Model outputs are parsed and normalized to one of the three predefined cell type labels. Quantitative performance was evaluated according to accuracy, precision, recall, and macro F1 score, enabling direct comparison with supervised CNN and hybrid CNN–transformer models.

While multimodal LLMs demonstrate promising interpretability through natural language explanations and reasoning traces, their quantitative performance remained limited in this setting. This limitation is attributed to the absence of domain-specific visual fine-tuning and the inherent difficulty of transferring general-purpose vision–language representations to specialized microscopy imagery. Accordingly, the LLM results are interpreted as exploratory benchmarks that contextualize the performance of learned visual representations under data-limited conditions.

### 3.4. Proposed Hybrid Transformer–CNN Model

To overcome the limitations of both standalone CNNs (local feature bias) and prompt-based LLM reasoning (lack of domain adaptation), we introduce a fine-tuned **HybridDeiT_EfficientNet_B7** architecture. This model synergistically combines the global semantic awareness of a distilled transformer (DeiT-Small) with the high-resolution morphological precision of a deep CNN (EfficientNet-B7).

#### 3.4.1. Rationale for Hybridization

The hybrid architecture explicitly leverages complementary inductive biases of CNNs and transformers. While the transformers capture global semantic relationships and long-range dependencies, which are critical for identifying distributed pathological cues, the CNNs capture the localized spatial textures and morphological granularity essential for fine cellular differentiation. This combination ensures that both global and local cues are utilized during training, leading to more robust classification in complex biomedical imagery.

#### 3.4.2. Architectural Design of the Proposed Model

The proposed hybrid architecture integrates two pretrained visual backbones that operate in parallel to jointly capture global contextual semantics and fine-grained cellular morphology.

The first branch employs DeiT-Small, a distilled vision transformer, to encode global semantic relationships through self-attention. Given an input microscopy image $I\in R^{H\times W\times C}$, the transformer produces a compact global embedding derived from the class token of the final encoder layer:

$$z_{t}\in R^{384}.$$

In parallel, the second branch utilizes **EfficientNet-B7** to extract hierarchical convolutional features that encode a localized texture and morphological structure. The convolutional feature maps

$$F_{c}=f_{\mathrm{CNN}}\left(I\right)$$

are aggregated using global average pooling (GAP) to obtain a compact visual descriptor:

$$z_{c}=\mathrm{GAP}\left(F_{c}\right)\in R^{2560}.$$

Feature fusion is performed at the embedding level through concatenation of the transformer and CNN representations:

$$z_{\mathrm{concat}}=\left[z_{t};z_{c}\right],$$

where [·;·] denotes vector concatenation.

The fused representation is subsequently mapped into a shared latent space using a learnable linear projection implemented as a fully connected layer:

$$z_{p}=Wz_{\mathrm{concat}}+b,$$

where $W\in R^{2048\times (384+2560)}$ and $b\in R^{2048}$ are trainable parameters that project the concatenated features into a 2048-dimensional embedding space.

The projected representation $z_{p}$ is then passed to a classification head consisting of a fully connected layer followed by Softmax activation, producing probabilities over the three Alzheimer’s-related cell types (astrocyte, cortical, and SH-SY5Y).

**Figure 4:**
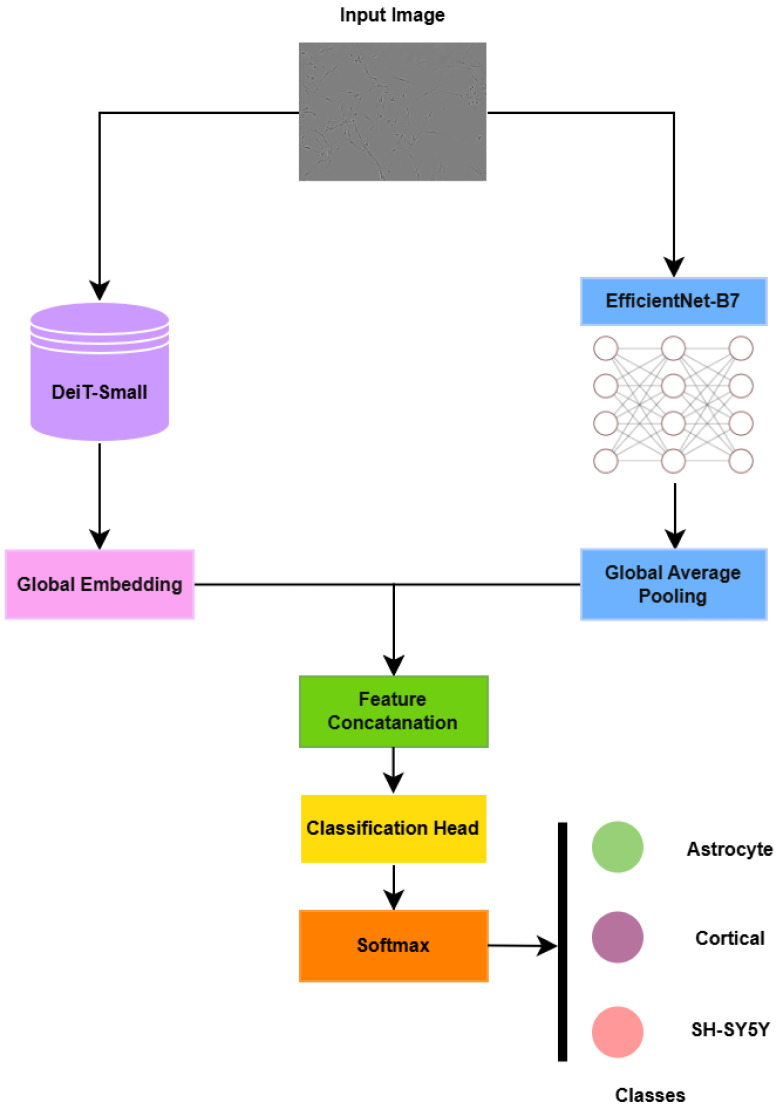
Proposed model’s architectural pipeline.

The overall architectural pipeline is illustrated in Figure 4.

#### 3.4.3. Training Configuration for Proposed Model

The training configuration we used for the proposed model is as follows:

- **Optimizer:** AdamW.
- **Scheduler:** Cosine annealing.
- **Loss Function:** Weighted CrossEntropyLoss to address class imbalance.
- **Batch Size:** 16.
- **Epochs:** 80.
- **Regularization:** Stochastic depth and label smoothing.
- **Data Augmentation:** Augmentations identical to the baselines are used—Random ResizedCrop, ColorJitter, GaussianBlur, and MixUp/CutMix—to maintain consistent preprocessing while increasing sample diversity.
- **Input Resolution:** DeiT path (224 × 224) and EfficientNet path (448 × 448–600 × 600). These images are downsampled separately for each pipeline to preserve the optimal receptive field for both networks.

### 3.5. Comparitive Evaluation Protocol

All models, comprising the deep learning baselines, multi-modal LLMs, and the proposed hybrid architecture, were evaluated under a consistent experimental protocol to ensure fairness and comparability. The evaluation metrics included the overall accuracy, macro F1 score, and per-class precision–recall curves, providing a comprehensive assessment of both global and class-specific performance. The dataset was partitioned into 70% for training, 15% for validation, and 15% for testing, maintaining stratified distribution across classes. To ensure reproducibility and statistical reliability, each experiment was repeated with three distinct random seeds, and the final reported results represent the averaged performance across these runs.

The methodological framework systematically contrasts three distinct learning paradigms—supervised CNNs, reasoning-based multi-modal LLMs, and a fine-tuned hybrid transformer–CNN model. Empirically, the HybridDeiT_EfficientNet_B7 achieved superior classification performance, demonstrating that explicit multi-modal feature fusion remains more effective than prompt-based inference in specialized biomedical image understanding tasks.

## 4. Results

### 4.1. Experimental Set-Up and Evaluation Metrics

All experiments were conducted using stratified fivefold cross-validation to ensure robust evaluation across different data splits. Class balancing via the SMOTE was applied *exclusively within the training portion of each fold*, while the validation and test sets consisted solely of original, unmodified microscopy images. This protocol ensured that no synthetic samples appeared in the evaluation data and eliminated any possibility of data leakage.

Following SMOTE-based rebalancing of the training folds, the effective training set size increased to 960 samples, while evaluation metrics were computed only on real images. Model performance was assessed using the accuracy, precision, recall, and macro F1 score. Particular emphasis was placed on the F1 score due to its balanced consideration of false positives and false negatives, which is especially important in medical imaging tasks, where misclassification can lead to misleading biological interpretations [48]. The experiments were conducted on an NVIDIA T4 GPU using PyTorch 2.9.0, CUDA 13.1, and Python 3.9.

### 4.2. Comparative Analysis of Methodological Approaches

#### 4.2.1. Prompt-Based Large Language Model Performance

Table 2 summarizes the performance of prompt-based multimodal large language models (LLMs) under zero-shot, few-shot, and chain-of-thought prompting strategies for Alzheimer’s-related cell type classification. These models were evaluated without task-specific fine-tuning and should therefore be interpreted as *prompt-only baselines* rather than optimized vision classifiers.

Across all prompting strategies, performance exhibited a clear hierarchy consistent with the model scale and architectural sophistication. GPT-5 achieved the highest accuracy range (50.94–58.82%), outperforming GPT-4o (41.98–45.32%) and Gemini 2.5 Flash (30.37–35.07%). Chain-of-thought prompting consistently improved performance for Gemini and GPT-5, suggesting that structured reasoning benefits complex visual categorization tasks. However, few-shot prompting yielded mixed results for GPT-4o, indicating sensitivity to prompt formulation.

The precision–recall trade-offs varied across the models; GPT-4o zero shot achieved relatively high precision (53.43%) at the expense of recall (44.37%), whereas GPT-5 with chain-of-thought prompting maintained a more balanced precision–recall profile (56.58% and 57.86%, respectively). Overall, while the prompt-based LLMs demonstrated encouraging zero- and few-shot reasoning capabilities, their performance remained constrained in this domain due to the absence of domain-specific visual adaptation.

#### 4.2.2. Fine-Tuned Deep Learning Model Performance

Table 3 presents the performance of fine-tuned deep learning models trained using ImageNet-pretrained weights. Across all CNN-only architectures, a consistent pattern emerged: validation accuracies remained moderate (approximately 64–66%), while test performance dropped sharply to near chance levels (accuracy of 32–35% and F1 score of about 31–35%). This discrepancy resulted in large generalization gaps exceeding 30% for most CNN architectures.

These results indicate that despite transfer learning, the standalone CNN models struggled to generalize effectively under extreme data scarcity and domain shifts from natural images to microscopy imagery. The consistency of this behavior across diverse architectures—including MobileNet variants, ResNet, DenseNet, InceptionNet, and EfficientNet-B7—suggests that the observed limitation is dataset-driven rather than architecture-specific.

In contrast, the transformer-based and hybrid models demonstrated substantially improved generalization. The DeiT-Small model alone achieved a test accuracy of 57.39% and F1 score of about 56.75% with a reduced generalization gap (15.72%), indicating that global self-attention mechanisms offer increased robustness under limited data conditions. The proposed hybrid DeiT-Small + EfficientNet-B7 architecture further improved performance, achieving the highest test accuracy (61.03%) and macro F1 score (61.85%). Compared with the CNN-only baselines, this represents a substantial improvement in precision–recall balance and overall robustness.

### 4.3. Architectural Analysis and Performance Trends

#### 4.3.1. Hybrid Architecture Advantages

The improved performance of the hybrid DeiT-Small + EfficientNet-B7 model can be attributed to complementary feature integration rather than reliance on a single architectural component. EfficientNet-B7 captures localized morphological and textural features relevant to cellular structure, while the DeiT-Small transformer models global spatial relationships and long-range dependencies within microscopy images.

The reduced generalization gap (17.48%) relative to CNN-only models (>31%) suggests that architectural diversity provides implicit regularization under SMOTE-balanced training. Attention mechanisms may help suppress spurious correlations amplified by oversampling, while convolutional features preserve a biologically meaningful local structure. Together, these components yield more stable representations under class imbalance.

#### 4.3.2. Cross-Methodological Performance Analysis

A comparison across all evaluated paradigms revealed distinct performance characteristics:

1. The **prompt-based LLMs** (30.37–58.82% accuracy) demonstrated variable performance, depending on the model scale and prompting strategy, but remained limited by the lack of domain-specific visual fine-tuning.
2. The **CNN-only models** (32.01–35.12% test accuracy) exhibited near-random performance under data scarcity, highlighting the limitations of conventional transfer learning for microscopy imagery.
3. The **transformer and hybrid models** (57.39–61.03% test accuracy) achieved improved generalization, with the hybrid CNN–transformer architecture providing the most consistent precision–recall balance.

**Figure 5:**
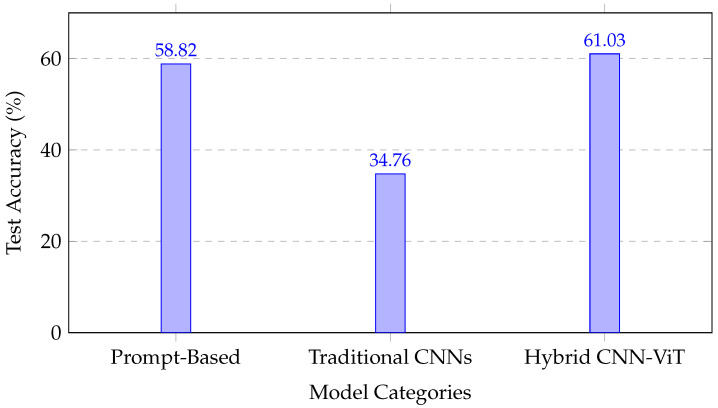
Performance comparison across methodological approaches, showing the superior performance of the hybrid CNN–transformer architecture.

Figure 5 illustrates the comparative performance across methodological categories, emphasizing the robustness gains achieved by integrating convolutional and attention-based representations.

#### 4.3.3. Statistical Significance and Clinical Relevance

The increase in the macro F1 score from approximately 33% for the CNN-only models to 61.85% for the hybrid architecture represents a substantial improvement in classification reliability under controlled SMOTE-balanced training. While these results do not yet meet the thresholds required for clinical deployment, they demonstrate meaningful gains in automated cell type discrimination relevant to exploratory Alzheimer’s disease research.

Importantly, all evaluation metrics were computed on original microscopy images, ensuring that the observed improvements reflect enhanced generalization rather than memorization of synthetic samples. Table 4 provides a consolidated summary of performance across all evaluated approaches.

The results position hybrid CNN–transformer architectures as a promising direction for cell-level Alzheimer’s disease image analysis under data-limited conditions, while underscoring the continued need for larger datasets and domain-specific pretraining to enable clinical translation.

## 5. Discussion and Limitations

This study presented a comparative evaluation of conventional convolutional neural networks (CNNs), prompt-based multimodal large language models (LLMs), and a hybrid CNN–transformer architecture for Alzheimer’s disease (AD) cell type classification using phase-contrast microscopy images. Among the evaluated approaches, the hybrid DeiT-Small + EfficientNet-B7 model achieved the strongest performance, attaining a test accuracy of 61.03% and a macro F1 score of 61.85 (Table 4). This constitutes a substantial improvement over standalone CNN baselines, whose F1 scores remained near chance levels (32.01–34.76%). These results indicate that coupling convolutional inductive biases with attention-based global reasoning yields more discriminative representations for fine-grained cellular image analysis, consistent with recent advances in hybrid vision architectures [49].

The observed performance gains can be attributed to complementary feature learning mechanisms. Convolutional layers effectively capture localized morphological characteristics such as texture, cell boundaries, and neuritic structures, whereas transformer-based self-attention enables modeling of long-range spatial dependencies and global contextual organization. This synergy is particularly well suited to microscopy imagery, where accurate classification depends on both cellular-scale morphology and broader spatial arrangements. Similar hybrid designs have demonstrated improved robustness and generalization across biomedical imaging tasks, further supporting the suitability of this architectural paradigm for cellular-level AD analysis [49,50].

In contrast, transfer learning from natural image datasets (e.g., ImageNet) proved inadequate for this task. The CNN-only baselines exhibited pronounced validation–test performance gaps exceeding 31%, indicating limited generalization under conditions of data scarcity and substantial domain shifts. This finding aligns with prior work demonstrating that ImageNet-pretrained representations often encode inductive biases poorly aligned with biomedical and microscopy image statistics [51,52]. Collectively, these results underscore the importance of domain-aware pretraining strategies, as emphasized in recent foundation-model research for pathology and neuroscience imaging [53,54].

The prompt-based multimodal LLMs achieved moderate performance (30.37–58.82% accuracy), with outcomes strongly influenced by the model scale and prompting strategy. While these models offer interpretability advantages through natural language reasoning, their quantitative performance remains limited in the absence of domain-specific visual adaptation. The difficulty of capturing subtle cellular morphology via text-guided inference alone likely contributes to this gap. Nevertheless, the observed performance ordering (GPT-5 > GPT-4o > Gemini 2.5 Flash) suggests that increasing the model capacity and improving multimodal alignment may enhance applicability to specialized biomedical tasks, consistent with broader trends in vision–language modeling [38].

Despite the relative advantages of the hybrid architecture, several limitations warrant consideration. First, the absolute classification accuracy (61.03%) remains substantially below thresholds typically required for clinical deployment, which often exceed 90% [43,55]. Second, although the synthetic minority over-sampling technique (SMOTE) was applied in a strictly controlled manner—restricted to feature space and training folds—the effective dataset size (960 samples) remains limited for training high-capacity transformer-based models. The persistent 17.48% validation–test gap suggests residual overfitting, reflecting constraints imposed by the limited sample diversity and partial reliance on synthetic balancing.

Additionally, this study was restricted to a single imaging modality and three cell types: astrocytes, cortical neurons, and SH-SY5Y neuroblastoma cells. While these classes are biologically relevant to AD pathology, the narrow scope limits the generalizability of the conclusions. Furthermore, the evaluation of multimodal LLMs was intentionally confined to prompt-based inference without weight adaptation to ensure fairness and reproducibility; however, this design choice precludes assessment of their full potential under domain-specific fine-tuning.

Overall, the findings indicate that effective AD cell type classification requires architectures explicitly tailored to biomedical imagery rather than direct repurposing of natural image models. Hybrid CNN–fransformer frameworks have emerged as a promising direction, as they integrate fine-grained cellular morphology with global spatial context, both of which are critical for modeling neurodegenerative disease mechanisms [46,56]. Future work should prioritize the expansion of microscopy datasets through multi-center collaborations, the development of microscopy-specific pretraining and augmentation strategies, and the integration of complementary modalities such as gene expression and protein biomarkers. Incorporating explainable AI (XAI) techniques will also be essential to improve interpretability and foster clinical trust in automated cellular analysis systems [57,58].

In summary, despite constraints related to dataset size and scope, this study provides a methodologically rigorous proof of concept demonstrating the advantages of hybrid CNN–transformer architectures for AD-related cell type classification. The results establish a foundation for future biologically informed and clinically relevant computational models while clearly delineating the challenges that must be addressed to advance toward real-world deployment.

## 6. Conclusions

This study presented a hybrid vision transformer–CNN framework for Alzheimer’s disease (AD) cell type classification from phase-contrast microscopy images. By integrating the global contextual modeling of a DeiT-Small transformer with the fine-grained morphological feature extraction of EfficientNet-B7, the proposed architecture demonstrated improved generalization compared with conventional CNN and standalone transformer baselines under data-limited conditions. The model achieved a test accuracy of 61.03% and a macro F1 score of 61.85, highlighting the advantages of combining convolutional and attention-based representations for cellular image analysis.

The results indicate that direct transfer learning from natural image datasets is insufficient for microscopy-based AD cell classification, whereas hybrid architectures better capture the complementary local and global visual cues required for this task. Prompt-based multimodal large language models, evaluated as zero- and few-shot baselines, provide interpretability benefits but remain limited in quantitative performance without domain-specific visual adaptation.

Despite these promising findings, the study was constrained by a modest dataset size, class imbalance, and a single imaging modality, which collectively limit clinical applicability. Accordingly, the presented results should be interpreted as a proof of concept rather than a deployable diagnostic system.

Future work will focus on expanding microscopy datasets through multi-center collaborations, exploring domain-specific pretraining strategies, incorporating additional biological modalities, and integrating explainable AI (XAI) techniques to improve interpretability and reliability. These directions are essential for advancing toward biologically grounded and clinically meaningful computational tools for Alzheimer’s disease research.

## Figures and Tables

**Table 1:** Recent state-of-the-art (SOTA) studies in Alzheimer’s disease classification.

Study	Key Challenges	SOTA Method	Data Type	Classification Result
EEM-AH and Machine Learning (2022) [41]	Segmentation accuracy, feature extraction	Enhanced Expectation Maximization Adaptive Histogram (EEM-AH)	AD MRI images	96.92% accuracy
Dual Attention-Aware Octave Convolution Network (2024) [42]	Early-stage AD detection, model efficiency	Attention mechanisms with octave convolutions	ADNI MRI dataset	99.87% accuracy
Pixel-Level Fusion with Vision Transformer (2023) [43]	Data integration, feature learning	Pixel-level image fusion with vision transformers	ADNI MRI and PET data	93.75% accuracy
Explainable Deep Learning with Multimodal Input Fusion (2023) [44]	Interpretability, multimodal data integration	Multimodal fusion	MRI and PET data	73.9% accuracy
Pareto Optimized Adaptive Learning (2023) [45]	Optimization of model parameters, image fusion	Pareto optimized VGG19 with transposed layer	ADNI MRI and PET data	80.2% accuracy
Optimized Convolutional Fusion for Multimodal Neuroimaging (2023) [46]	Data integration, feature extraction from multiple modalities	Optimized fusion of multiple neuroimaging modalities	AANLIB dataset	99% accuracy

**Table 2:** Performance comparison of prompt-based LLM methods.

Model	Prompting Strategy	Accuracy (%)	F1 Score	Precision	Recall
Gemini 2.5 Flash	Zero Shot	30.37	27.54	24.17	32.01
Gemini 2.5 Flash	Few-Shot	31.90	28.39	25.11	32.75
Gemini 2.5 Flash	Chain of Thought	35.07	30.51	26.21	36.24
GPT-4o	Zero Shot	41.98	48.48	53.43	44.37
GPT-4o	Few Shot	43.15	40.11	42.73	37.79
GPT-4o	Chain of Thought	45.32	47.64	48.92	46.43
GPT-5	Zero Shot	50.94	51.64	52.18	51.11
GPT-5	Few Shot	53.51	53.23	54.21	52.29
GPT-5	Chain of Thought	58.82	57.21	56.58	57.86

**Table 3:** Performance comparison of fine-tuned models.

Model Architecture	Validation Accuracy (%)	Test Accuracy (%)	Test F1 Score	Generalization Gap (%)
MobileNet	64.93	32.74	31.62	32.19
MobileNetV2	66.21	34.76	33.97	31.45
ResNet	65.15	32.01	32.64	33.14
InceptionNet	64.85	33.82	34.16	31.03
DenseNet	64.75	33.53	33.01	31.22
EfficientNet-B7	62.61	35.12	35.01	27.49
DieT-Small	73.11	57.39	56.75	15.72
DieT-Small + EfficientNet-B7	78.51	61.03	61.85	17.48

**Table 4:** Summary of key performance metrics across all approaches. Bold indicates our model.

Approach Category	Best Accuracy (%)	Best F1-Score	Avg. Gen. Gap (%)	Improvement vs. CNN
Prompt-Based Methods	58.82	57.21	N/A	+69.3%
Traditional Fine-Tuned CNNs	34.76	34.16	31.80	–
**Hybrid CNN–Transformer**	61.03	61.85	**17.48**	**+75.6%**

## Data Availability

The data that support the findings of this study are available upon reasonable request.
